# Attention-enhanced multi-task learning for binary segmentation and fine-grained aquatic plant classification in UAV imagery

**DOI:** 10.1038/s41598-026-51881-5

**Published:** 2026-05-22

**Authors:** Ashifur Rahman, M. M. Mahbubul Syeed, Razib Hayat Khan, Kaniz Fatema, Tarem Ahmed, Safiqul Islam

**Affiliations:** 1 Department of Computer Science and Engineering, Independent University, Bangladesh, Dhaka 1229 Bangladesh; 2 RIoT Research Center, Independent University, Bangladesh, Dhaka 1229 Bangladesh; 3 Department of Computer Science, Oslo Metropolitan University, Oslo, Norway

**Keywords:** Computational biology and bioinformatics, Ecology, Ecology, Environmental sciences, Mathematics and computing

## Abstract

Accurate monitoring of aquatic vegetation from unmanned aerial vehicle (UAV) imagery remains challenging due to complex water backgrounds, severe inter-class similarity, and the lack of balanced, dual-annotated datasets. Existing studies primarily address segmentation or classification independently, limiting their effectiveness for integrated species-level analysis. To address these gaps, this study proposes a clearly defined attention-enhanced multi-task learning framework that simultaneously performs binary segmentation and 14-class species classification, enabling unified structural and semantic understanding. The model employs a shared encoder with attention-guided skip connections and a joint optimization strategy to enhance feature discrimination while reducing redundancy. Comprehensive ablation analysis demonstrates that attention improves both segmentation and classification performance, while joint learning with Gaussian blur achieves the best overall balance, confirming the complementary role of spatial and semantic features. On a newly collected UAV dataset from diverse wetlands in Bangladesh, the proposed model achieves a Dice coefficient of 0.7344, mIoU of 0.6904, and pixel accuracy of 0.8757 for segmentation, along with 98.77% classification accuracy and an F1-score of 0.9874, indicating strong performance across both tasks. In addition, computational complexity analysis shows that the proposed framework reduces parameters by $\sim$50% (31.10M vs. 62.09M), lowers FLOPs (54.66 vs. 96.31 GFLOPs), and improves inference speed by $\sim$48.6% compared to deploying separate single-task models for segmentation and classification, demonstrating its suitability for real-time UAV deployment. Furthermore, Gradient-weighted Class Activation Mapping (Grad-CAM) and Grad-CAM++ are employed to provide visual explanations of model predictions, improving interpretability and reliability. The results demonstrate robust performance in complex aquatic environments and highlight the framework’s suitability for large-scale biodiversity monitoring, invasive species detection, and data-driven freshwater ecosystem management.

## Introduction

Aquatic vegetation plays a crucial role in maintaining water quality, providing habitat, and supporting biodiversity in freshwater ecosystems^1,2^. However, rapid environmental change and increasing human pressures demand scalable, high-resolution monitoring tools that go beyond sparse field surveys. Recent advances in unmanned aerial vehicles (UAVs) and imaging sensors enable efficient acquisition of fine-grained spatial data over large water bodies, but the resulting imagery poses significant challenges due to complex backgrounds, water reflections, and high inter- and intra-class similarity among plant species^2,3^. These challenges make accurate species-level mapping particularly difficult, especially when subtle morphological differences must be distinguished under varying illumination and environmental conditions.

Deep learning (DL) has become the de facto standard for visual recognition in remote sensing, including vegetation mapping and aquatic ecosystem analysis^4–6^. Convolutional encoder–decoder architectures such as U-Net and its variants have demonstrated strong performance for semantic segmentation of high-resolution imagery^7,8^, while classification-focused models achieve high accuracy in controlled plant recognition tasks. More recently, multi-task learning (MTL) has been introduced to jointly solve related problems such as segmentation, classification, and detection, improving representation learning through shared feature extraction. For example, MultiTaskDeiT-UNet^9^ combines transformer-based classification with segmentation but struggles in dense mixed-species environments, while MTBD-Net^10^ integrates attention modules for biofouling analysis but is limited to single-site evaluation and exhibits moderate segmentation accuracy (Dice 59.46%). Similarly, MTLSegFormer^11^ leverages transformer-based cross-task attention but introduces high computational cost and task dependency constraints. Other works such as MTJNet^12^ and PDLC-ViT^13^ report strong classification performance; however, they are primarily evaluated on controlled or domain-specific datasets, limiting real-world generalization.

Despite these advances, several critical gaps remain. First, most existing approaches either focus on homogeneous agricultural settings or controlled benchmarks, lacking robustness to complex aquatic environments characterized by occlusion, water reflections, and heterogeneous plant distributions. Second, many MTL frameworks emphasize either classification or segmentation performance without explicitly analyzing the mutual benefits of coupling pixel-level and image-level supervision. Third, prior UAV-based aquatic studies often rely on specialized sensors (e.g., hyperspectral imaging^14^) or computationally intensive pipelines^15,16^, which limit scalability and real-time applicability. Furthermore, evaluation is frequently restricted to single geographic locations or small datasets, reducing confidence in model generalization and robustness. Finally, although some works incorporate explainability tools such as Grad-CAM, these analyses are typically qualitative and lack systematic evaluation of model reliability.

In this paper, an attention-enhanced multi-task learning framework is proposed for simultaneous binary segmentation and fine-grained aquatic plant classification in UAV imagery acquired over multiple sites. It uses attention-guided decoding to fuse global semantic and local structural cues, with joint optimization over a segmentation head and a 14-class classification head. Unlike prior works, the proposed approach is specifically designed to operate under real-world UAV conditions using standard RGB imagery, avoiding reliance on expensive sensors while maintaining high discriminative capability in visually complex aquatic scenes. In addition, the framework explicitly investigates the synergy between segmentation and classification tasks, demonstrating how shared representations improve both localization and species recognition under class imbalance.

On the collected dataset, the proposed network achieves a Dice coefficient of 0.7344, a mean IoU of 0.6904, and a pixel accuracy of 0.8757 for segmentation while obtaining 98.77% accuracy, 0.9872 precision, 0.9883 recall, and 0.9874 F1-score for species classification, demonstrating robust performance under challenging imaging conditions. To address the limitations of prior qualitative-only explainability studies, this work further incorporates a more structured analysis using Grad-CAM^17^ and Grad-CAM++^18^, including comparisons across correct and incorrect predictions and assessment of class-specific attention consistency, thereby strengthening the reliability and interpretability of the proposed model for ecological monitoring and decision support.

The main contributions of this work is summarized below.

- Proposes an attention-enhanced multi-task deep learning architecture that concurrently performs binary segmentation and fine-grained species classification using a shared encoder and task-specific heads, explicitly exploiting the complementary pixel-level and image-level information.
- The dataset is newly curated and includes 14 aquatic plant species, both rare and common, collected from different wetlands across Bangladesh under varied natural conditions. It consists of a total of 1761 segmentation samples and 1468 classification samples. The dataset is approximately balanced across classes, making it suitable for effective multi-task learning and evaluation.
- A comprehensive experimental study comparing single-task and multi-task training, evaluating segmentation and classification metrics, and demonstrating the effectiveness of attention-guided feature fusion for aquatic vegetation analysis and its potential for large-scale monitoring and management.
- The proposed model reduces parameters by approximately 50% (31.10M vs. 62.09M), lowers computational cost in terms of FLOPs (54.66 vs. 96.31 GFLOPs), and improves inference speed by around 48.6%, demonstrating higher efficiency compared to separate single-task models.
- An interpretability analysis using Grad-CAM and Grad-CAM++ is performed to provide visual explanations of classification decisions, enhancing model transparency and reliability for real-world aquatic ecosystem monitoring applications.

## Related work

The convergence of deep learning, UAV imaging, and multi-task learning has generated substantial research in automated vegetation monitoring, plant classification, and semantic segmentation. Relevant prior work can be grouped into four themes: (i) MTL frameworks jointly solving classification and segmentation for plant targets; (ii) attention-enhanced MTL architectures for segmentation; (iii) UAV-based aquatic vegetation mapping; and (iv) super-resolution reconstruction (SRR) combined with semantic segmentation. Recent research has explored the potential of integrating deep learning, UAV imaging, and MTL for classification and semantic segmentation tasks to automate vegetation monitoring. A synopsis of these works is presented in Table 1.

### Multi-task learning for plant classification and segmentation

A recurring finding across the MTL literature is that shared feature representations consistently outperform independently trained single-task models. Vinod et al.^9^ introduced MultiTaskDeiTUNet, coupling a Data-efficient Image Transformer (DeiT) classification backbone with a U-Net segmentation branch under a single encoder for simultaneous tree plantation density classification and crown segmentation in satellite imagery. The model achieved a mean F1-score of 0.91 for density classification and a mean IoU of 0.73 for crown segmentation, with ablation confirming that freezing DeiT weights degraded the classification F1-score to 0.48, underscoring the necessity of joint end-to-end training. However, performance deteriorated in high-density mixed-species stands, and scalability to national-level mapping remains limited by computational cost and the absence of multispectral or LiDAR integration. Sharma et al.^12^ similarly validated cross-task feature sharing through MTJNet, a network with a shared encoder and task-specific heads for concurrent medicinal plant-species (accuracy 98.7%) and leaf (accuracy 97.4%) classification, though the study was restricted to medicinal species and did not address real-world challenges such as occlusion or variable illumination.

In the crop disease domain, Luan et al.^19^ proposed ALMDR, which jointly performs multi-label disease classification, lesion detection, and lesion segmentation in apple leaves using a Group Feature Pyramid Network and dual segmentation heads, attaining a classification F1-score of 93.74%, detection mean average precision (mAP) of 51.32%, and severity regression coefficient of determination ($R^2$) of 0.9757, outperforming YOLOv9 while operating at near-real-time speeds. Zhencun and Dong^20^ demonstrated a simpler multi-task VGG16 model, achieving 97.22% and 98.75% accuracy on rice and wheat leaf disease recognition, respectively, though the very small dataset (40 images per class) and absence of precision/recall/F1-score reporting limit the interpretability of the results. More rigorously, Hemalatha et al.^13^ introduced PDLC-ViT, a Vision Transformer-based MTL model employing co-scale, co-attention, and cross-attention mechanisms for simultaneous plant disease localization and classification, setting state-of-the-art results on PlantVillage with 99.97% accuracy and Grad-CAM-based interpretability. However, the controlled benchmark setting raises concerns about generalization to complex natural environments, particularly aquatic ecosystems, and the authors note that reliance on basic feature map concatenation restricts deeper cross-task feature interaction.

### Attention-enhanced multi-task architectures for segmentation

Attention mechanisms provide a powerful complement to MTL by selectively amplifying discriminative features in visually complex domains. Cui et al.^10^ proposed MTBD-Net for marine biofouling assessment, employing a ResNet-50 backbone augmented with a Feature Pyramid Network and Convolutional Block Attention Module (CBAM) to jointly classify, detect, and segment biofouling organisms, outperforming single-task U-Net (Dice 52.62%) and DeepLabV3 (Dice 54.74%) baselines with a Dice of 59.46%. Despite this, persistent confusion between adjacent severity classes and evaluation confined to a single marine site constrain its generalizability. Goncalves et al.^11^ extended SegFormer with a multi-task decoder (MTLSegFormer) and Transformer-based cross-task attention to jointly detect crop lines, planting gaps, and defoliation from UAV imagery, improving gap segmentation F1-score from 0.7115 to 0.7478 over the single-task baseline. The architecture’s computational overhead makes edge deployment challenging, and the cross-task attention benefit was found to be task-dependency specific, highlighting that attention-guided feature fusion must be carefully designed to generalize across dissimilar task pairs.

### UAV-based aquatic vegetation monitoring

UAVs have grown increasingly prominent for aquatic and wetland ecosystem monitoring owing to their high spatial resolution and operational flexibility. Yu et al.^14^ developed SpectralUFormer for aquatic plant classification in crab ponds, combining a channel attention block encoder for UAV hyperspectral imagery with a cascaded global-local MLP decoder, achieving 93.15% overall accuracy and a kappa coefficient of 89.14%. However, the reliance on expensive hyperspectral sensors, information loss from simple upsampling, and weaker per-class performance for spectrally similar species such as Elodea nuttallii limit its practical scalability. From a survey-methodology perspective, Chen et al.^21^ showed that oblique drone photogrammetry with gimbal pitch angle $\theta < 55^\circ$ avoids sun glint and extends the operational seagrass mapping window by 40 minutes in sunny conditions, though the oblique approach increased reprojection error by 10.9%, and the study was validated at a single site. These findings collectively underscore that RGB-based UAV imaging of aquatic vegetation is highly sensitive to background variability, reinforcing the need for robust learned feature representations.

### Super-resolution reconstruction and semantic segmentation

Low spatial resolution from consumer-grade UAVs constrains pixel-level classification accuracy, motivating the integration of super-resolution reconstruction (SRR) as a preprocessing stage. Zhao et al.^16^ coupled MambaIR-based SRR with a Mamba segmentation model for blueberry maturity assessment and achieved a segmentation mean IoU of 83.15% at 4$\times$ magnification, which was identified as the optimal scaling factor before performance saturation. However, their results also showed that noise can significantly degrade performance, with mIoU reductions ranging from 24.2% to 41.5%, highlighting sensitivity to adverse imaging conditions. Similarly, Tao et al.^15^ conducted one of the first studies integrating SRR with semantic segmentation for tobacco weed–crop differentiation, evaluating CNN, Transformer (DPT+DINOv2 achieving 90.75% mIoU), and Mamba-based architectures, and again confirming 4$\times$ magnification as the most effective setting. These studies demonstrate that SRR can substantially enhance downstream segmentation accuracy, although the additional computational cost limits its practicality for real-time UAV-based deployment. A consolidated comparison of all reviewed studies, including model architectures, dataset characteristics, key contributions, quantitative results, and identified limitations, is presented in Table 1.

**Table 1:** Summary of multi-task learning and UAV-based agricultural vision studies.

Ref.	Year	Model / Method	Dataset	Key Contribution	Results	Limitations
^9^	2024	MultiTaskDeiT UNet	Satellite imagery	Joint DeiT classification + U-Net segmentation in unified MTL encoder	F1-score: 0.91 (density); mIoU: 0.73 (crowns)	Struggles with high-density mixed-species areas; no multispectral/LiDAR integration
^10^	2025	MTBD-Net (ResNet-50 + FPN + CBAM)	Biofouling Dataset (BFD)	Unified MTL for biofouling classification, detection, and segmentation with CBAM attention	Dice: 59.46%; mAcc: 84.22%; mF1: 84.10%	Single-site evaluation; confusion between Moderate and Heavy severity classes
^12^	2024	MTJNet (shared encoder + dual heads)	Medicinal plant benchmark	Joint plant-species and leaf classification via shared encoder with task-specific heads	Plant accuracy: 98.7%; Leaf accuracy: 97.4%	Restricted to medicinal plants; occlusion and illumination not addressed
^14^	2024	SpectralUFormer (CAB + G-L MLP decoder)	UAV hyperspectral imagery, crab ponds	Hybrid spectral-spatial encoder and cascaded MLP decoder for aquatic plant classification	Overall Accuracy: 93.15%; Kappa: 89.14%	Expensive hyperspectral sensors; weaker results for spectrally similar species
^11^	2023	MTLSegFormer (SegFormer + MTL decoder)	Crop line/gap & soybean defoliation datasets (320 imgs)	Transformer cross-task attention for correlated precision-agriculture segmentation tasks	Gap F1-score: 0.7478 (vs. 0.7115 baseline)	Computationally heavy; benefit is task-dependency specific
^19^	2025	ALMDR (GFPN + MLCH + dual seg. heads)	Apple leaf disease dataset	MTL for multi-label disease classification, lesion detection, segmentation, and severity estimation	F1-score: 93.74%; Det. mAP: 51.32%; R$^2$: 0.9757	Apple-only; overlapping lesion feature entanglement; not optimised for edge devices
^20^	2021	Multi-task VGG16 (transfer learning)	Rice and wheat leaf disease (40 imgs/class)	Shared VGG16 backbone for simultaneous rice and wheat disease recognition	Rice: 97.22%; Wheat: 98.75%	Very small dataset; accuracy only reported; no cross-validation
^13^	2024	PDLC-ViT (ViT + co/cross-attention)	PlantVillage dataset	ViT-based MTL for plant disease localisation and classification with Grad-CAM explainability	Accuracy: 99.97%; MAP: 99.18%; MAR: 99.11%	Controlled benchmark only; basic feature concatenation limits cross-task depth
^21^	2025	Oblique drone photogrammetry	Futtsu Tidal Flat, Tokyo Bay	Theoretical model linking gimbal pitch angle to sun glint avoidance for seagrass mapping	Mapping window +40 min; reprojection error +10.9%	Single-site validation; accuracy-coverage trade-off at $\theta > 55^\circ$
^16^	2025	MambaIR SRR + Mamba segmentation	UAV blueberry field imagery	SRR pre-processing pipeline to enhance low-resolution UAV images before maturity segmentation	mIoU: 83.15% at 4$\times$ magnification	Noise-sensitive (mIoU drop 24–41%); computationally heavy; motion blur unaddressed
^15^	2025	SRR (RCAN) + Transformer (DPT+DINOv2)	Tobacco field UAV dataset (annotated)	First SRR + semantic segmentation study for UAV-based weed-crop differentiation in tobacco fields	Best mIoU: 90.75%; PSNR: 24.98 dB; SSIM: 69.48%	High-fidelity models not real-time deployable; limited endurance of consumer UAVs

### Research gaps and positioning of the proposed framework

The reviewed literature consistently demonstrates that MTL outperforms single-task alternatives for vegetation analysis^9,12,13,19,20^, that attention mechanisms enhance feature discrimination in complex imaging conditions^10,11^, and that UAV-based approaches face unique challenges from background variability and sensor limitations in aquatic environments^14–16,21^. However, no prior study has coupled binary segmentation with fine-grained species-level classification specifically for freshwater aquatic plant monitoring from UAV RGB imagery. Existing aquatic vegetation studies rely on costly hyperspectral sensors^14^ or address only survey-level photogrammetric optimization^21^, while the class imbalance problem and the lack of dual-annotated balanced datasets remain unresolved. Furthermore, attention-guided skip connections within a shared encoder—rather than applied solely in the classification branch or decoder—have not been explored for this task, and interpretability tools such as Grad-CAM have been applied only sporadically in single-domain plant studies^13^. The proposed attention-enhanced multi-task framework addresses these gaps through a shared encoder architecture with attention-guided skip connections, a jointly optimized global classification branch, a newly curated balanced UAV dataset of 14 aquatic species from Bangladeshi wetlands, and integrated Grad-CAM and Grad-CAM++ explainability analysis.

## Methodology

**Fig. 1:**
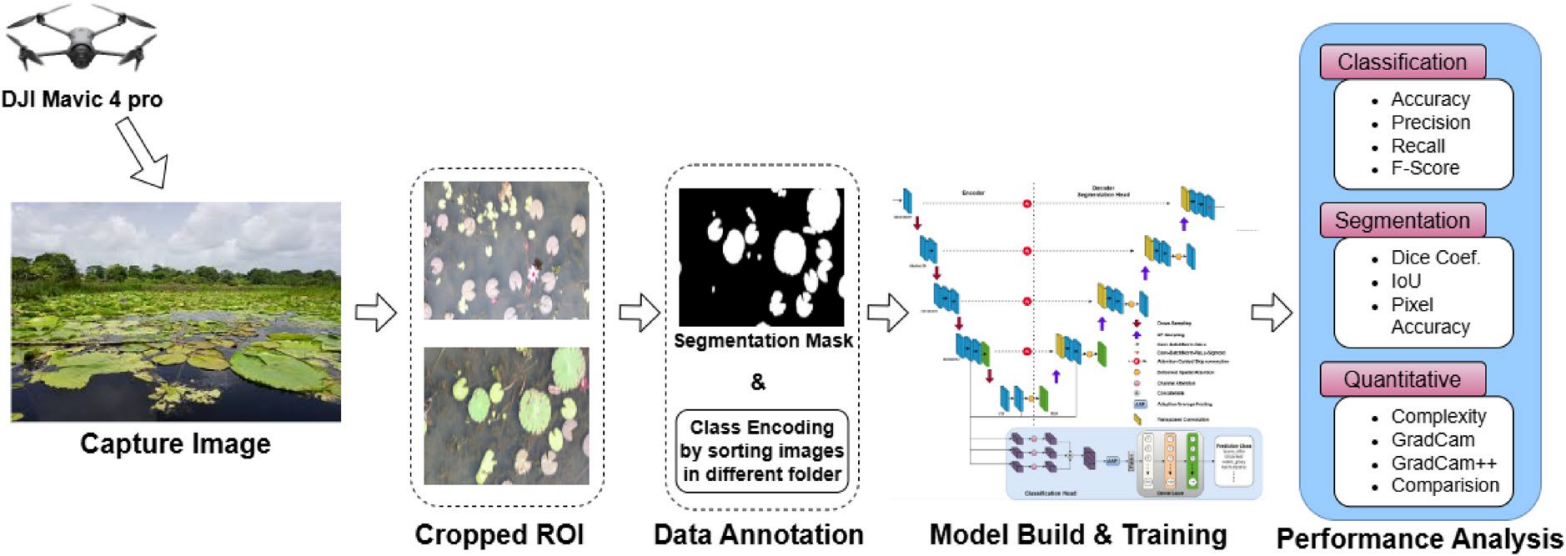
Steps followed in Conducting this Research.

This study follows a systematic and structured methodological framework to support the rigorous, unbiased, and technically validated development of a multi-task deep learning model for the assessment of aquatic plants. The adopted workflow is organized into a series of sequential steps to ensure clarity, reproducibility, and methodological consistency. An overview of this framework is presented in Fig. 1 and a detailed discussion is presented in the following sections.

### Data preparation

#### Data acquisition

Bangladesh hosts an extensive wetland landscape, including rivers, floodplains, haor basins, beels, ponds, and seasonal wetlands, that support more than 300 species of aquatic and semi-aquatic plants. For this study, aquatic plant data are collected from three prominent sites, namely, (a) Aquatic Museum ($\hbox {N}24^{\circ }43'20.9''\,\hbox {E}90^{\circ }25'56.0''$) and Botanical Garden ($\hbox {N}24^{\circ }43'29.9''\,\hbox {E}90^{\circ }26'29.3''$) of Bangladesh Agricultural University (BAU), Mymensingh, (b) Zinda Park ($\hbox {N}23^{\circ }51'56.8''\,\hbox {E}90^{\circ }31'5.2''$), Dhaka and (c) Water Lily Lake (Shapla Bill, $\hbox {N}23^{\circ }52'31.3''\, \hbox {E}90^{\circ }31'24.6''$), Dhaka. Among these locations, the BAU site hosts the largest live collection of aquatic plant species, while the other two sites cover approximately 50 acres of natural lakes and shallow wetland consisting of aquatic plant vegetation.

To capture images a DJI Mavic 4 Pro drone equipped with Hasselblad triple camera system is used. The image resolution is set to 1920 $\times$ 1080 px (HD), and ISO, shutter speed, aperture, and focal length are set to auto-mode for dynamic adjustment in different environmental conditions such as sunlight, reflection, and shadows. Furthermore, burst mode is used for the continuous capture of high-quality images. To reduce angular vibration and motion blur for optimal image quality, the camera is pointed directly downward and perpendicular to the flight path. The cruising speed is maintained at 0.1 m/s with an altitude of 2.5 m above the water surface, providing a ground sampling distance (GSD) of 0.04–0.05 cm/px. These settings ensure a stable flight cruise, minimize over/under exposure, and avoid water rippling due to propeller thrust. The large ROI is divided into several non-overlapping segments, and DJI waypoints are set for comprehensive coverage of the entire region.

A data collection campaign was conducted over multiple days using a drone under ideal weather conditions with bright light and little or no wind. The collected images were manually inspected to exclude those affected by motion blur or poor exposure, and reshoots were performed when necessary. The resulting raw dataset of approximately 1000 images covering 14 aquatic plant species was carefully evaluated to ensure data quality prior to further processing. After this quality assessment, regions of interest (ROI) were manually cropped and refined to ensure high annotation consistency. Following ROI extraction and preprocessing, a total of 1761 segmentation samples and 1468 classification samples were obtained and used for the final experiments.

#### Image annotation for semantic segmentation

**Fig. 2:**
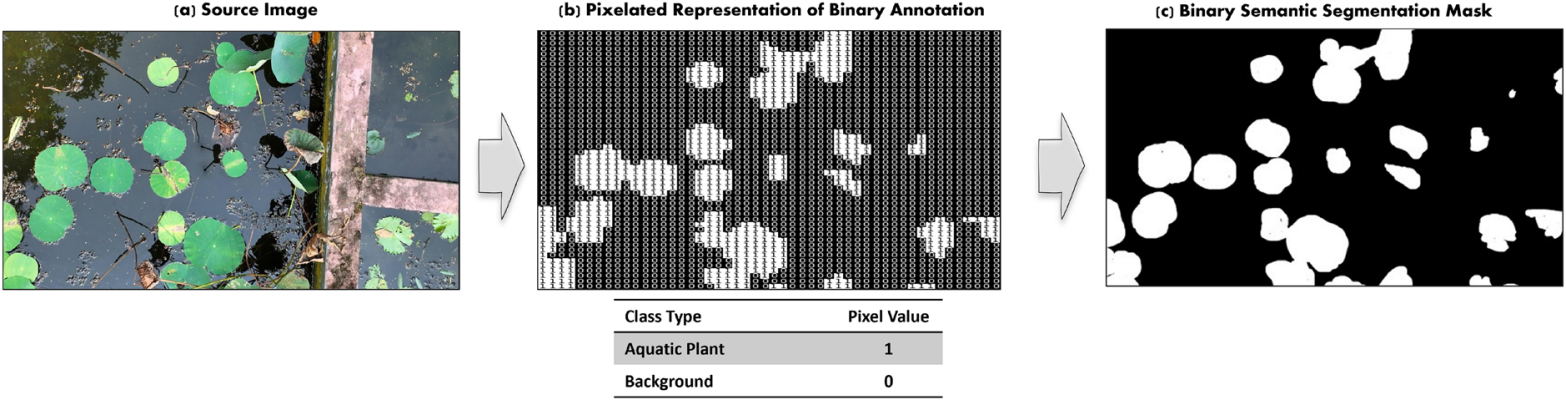
Image Annotation Process for Semantic Segmentation.

Each image is annotated for binary segmentation with pixel-level delineation. To carry out this annotation process for each UAV image, all the pixels that fall within identified plant leaves are annotated with pixel value 1 (one) and the rest of the pixels (i.e., the background) are annotated with pixel value 0 (zero). For precision annotation, the *DigitalSreeni Image Annotator tool*$,$ and *Wacom One DTC133 (13.3-inch Full-HD)* with precision drawing pen (having pressure sensitivity level of 4,096) is used. A representative example of this entire annotation process is presented in Fig. 2.

#### Image processing for classification

**Fig. 3:**
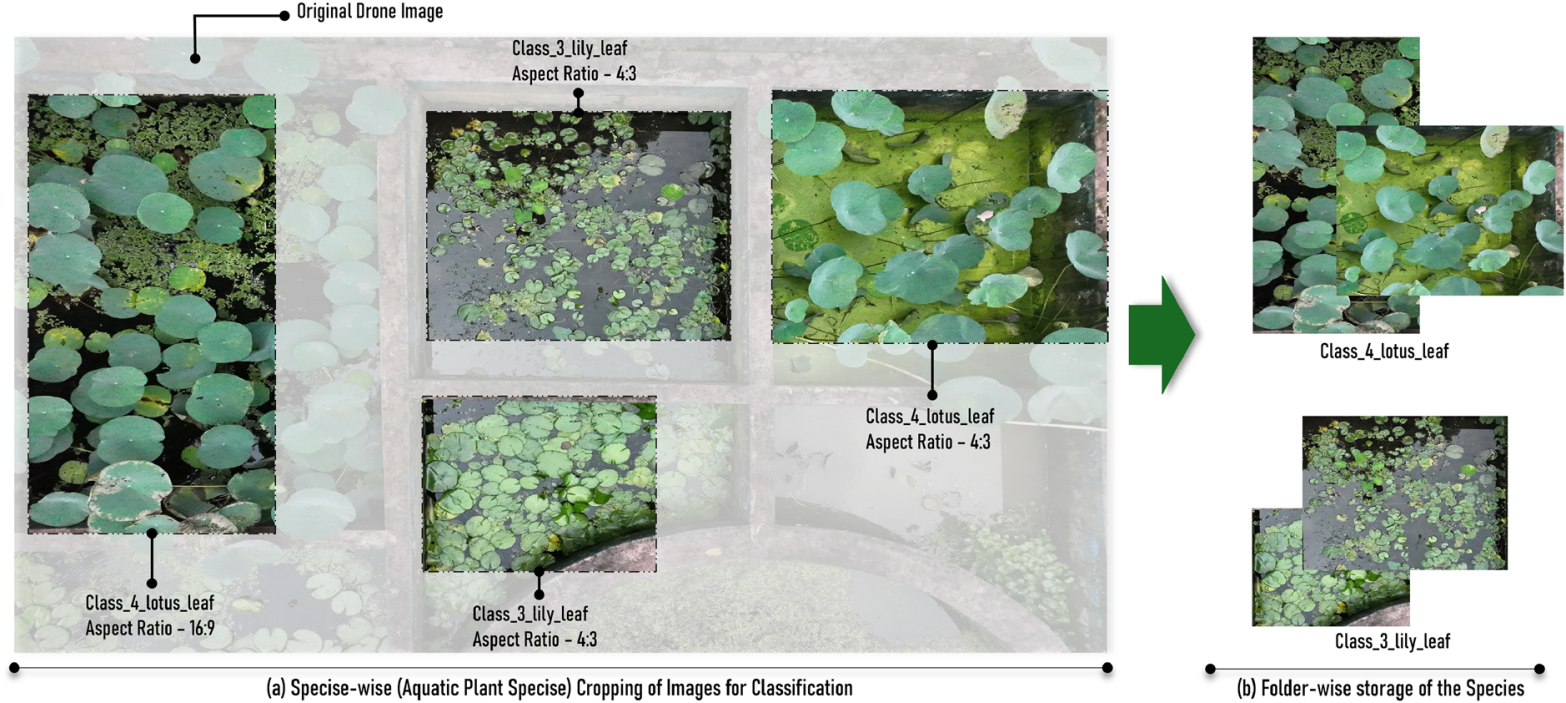
Cropping and Labeling of Aquatic Plant Species for Classification Task.

To perform the classification task, each UAV image is cropped to isolate individual aquatic plant species present in that image and categorized into species-wise folders. This is due to the fact that large water repositories (e.g., lakes and water reservoirs) host multiple types of aquatic plants. While the drone flies at a height of 2.5 m above the water surface, it captures approximately 0.5 sqm of area in a single frame that contains multiple plant species. To perform the cropping process, the expert annotators manually examined each image to isolate individual plant species that are visually separable (i.e., there is no or minimal overlap) and then cropped them, maintaining the following aspect ratios: 16:9 and 4:3. Moreover, the cropped areas are kept large enough to retain structural details without introducing distortion or loss of texture. A representative sample of this cropping process is illustrated in Fig. 3.

The overall dataset distribution used in this study is summarized in Table 2, which presents the number of training and testing samples for both segmentation and classification tasks. The segmentation dataset consists of 1561 training and 200 testing image-mask pairs, while the classification dataset includes 1062 training and 406 testing samples. This balanced split ensures sufficient representation for both tasks and enables reliable evaluation of the proposed multi-task framework.

**Table 2:** Segmentation and classification task train and test dataset distribution.

Task	Train Data	Test Data	Total
Segmentation	1561	200	1761
Classification	1062	406	1468

**Fig. 4:**
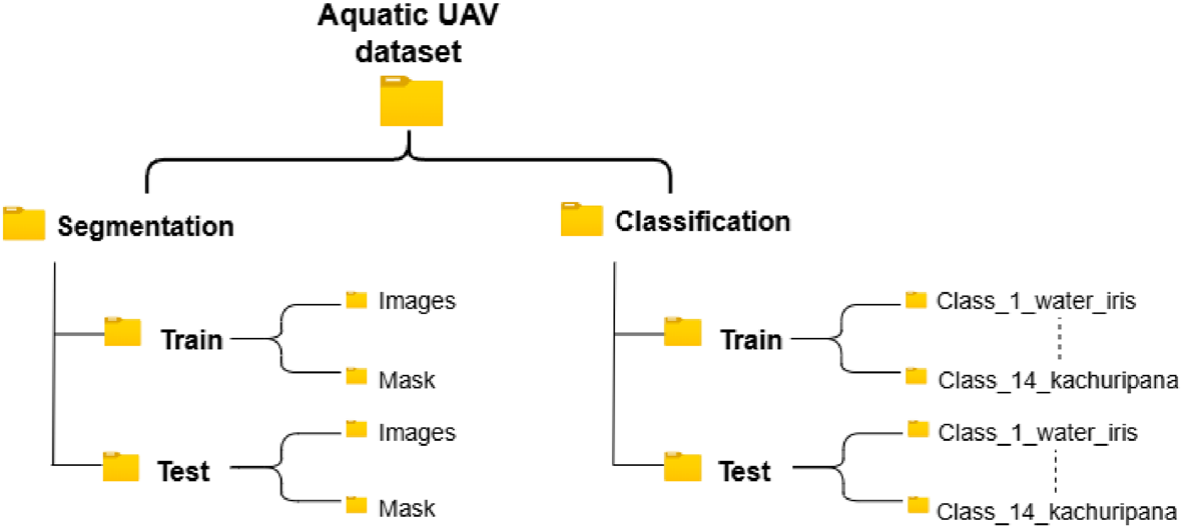
Overview of the Aquatic UAV dataset organization showing task-wise separation into segmentation and classification branches with corresponding train-test splits and class-wise structure.

The organizational structure of the dataset is illustrated in Fig. 4. The dataset is first organized by task into segmentation and classification directories. In the segmentation branch, the data is divided into training and testing subsets, where each subset contains separate folders for images and their corresponding binary masks, ensuring proper pairing between input images and ground-truth annotations for supervised learning. The segmentation dataset follows a $\sim 85\%$ training and $\sim 15\%$ testing split, enabling sufficient pixel-level learning while preserving a reliable evaluation set. In the classification branch, images are further grouped into species-specific folders within the training and testing sets. The classification dataset follows a $\sim 65\%$training and $\sim 35\%$ testing split with no overlap between images in these subsets, ensuring unbiased evaluation and preventing data leakage. The use of different data splits for segmentation and classification is intentional, as it reflects task-specific requirements and helps maintain a balanced distribution of samples across both training and testing sets. Each class folder corresponds to a distinct aquatic plant species (e.g., water iris, lily leaf, lotus leaf, water poppy, kachuripana, etc.), ensuring clear separation of categories and facilitating efficient supervised learning. This structured arrangement also supports scalable dataset management and simplifies data loading during model training. This dataset is publicly available at the Mendeley^22^ data repository.

**Fig. 5:**
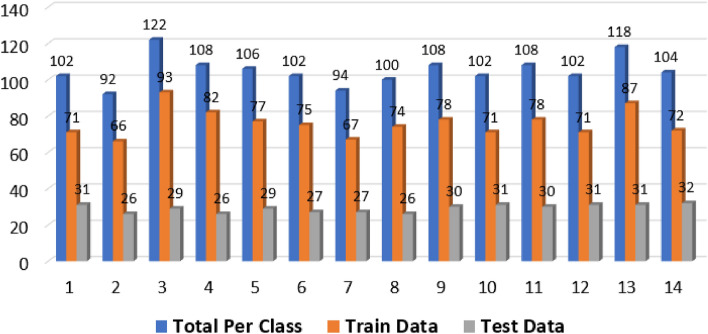
Class-wise data distribution for the classification task.

Furthermore, the class-wise data distribution of the classification dataset is visualized in Fig. 5. The figure highlights the number of total samples per class (blue), training samples (orange), and testing samples (gray) across all 14 species. Although minor variations exist between classes, the dataset is relatively well-balanced compared to typical ecological datasets, which often suffer from severe class imbalance. This balanced distribution helps reduce model bias toward dominant species and improves generalization performance across less-represented classes. Additionally, the consistent proportion between training and testing samples for each class ensures fair evaluation and prevents skewed performance metrics.

### Data preprocessing for model training and testing

All UAV images and corresponding segmentation masks are resized to a fixed spatial resolution of $256 \times 256$ pixels to ensure consistent input dimensions and stable training across both segmentation and classification tasks. A detailed description of the dataset is provided in the previous subsection. Pixel intensities are then scaled to the range $[0,1]$ and normalized using a mean of 0.5 and a standard deviation of 0.5 to center the data distribution and improve optimization stability during training:

1 $$\begin{aligned} \hat{x} = \frac{x - 0.5}{0.5}. \end{aligned}$$

To improve robustness against noise and illumination inconsistencies inherent in UAV imagery, a Gaussian blur with kernel size 3 and $\sigma = 1.0$ is applied to the input images. This preprocessing step acts as a low-pass filtering operation that suppresses high-frequency noise such as sensor artifacts and minor lighting variations, while preserving important structural patterns like vegetation boundaries and spatial context. This is particularly beneficial in aerial imaging conditions where environmental variability is high, thereby improving the generalization capability of the model^23,24^.

For the classification task, the dataset is organized in a class-wise directory structure as mentioned in Fig. 4, where each subfolder corresponds to a distinct aquatic plant species. Class labels are automatically extracted from folder names and mapped into integer indices using a deterministic encoding scheme. Specifically, all class names are sorted alphabetically, and each class is assigned a unique integer label via a class-to-index mapping, while an inverse mapping is maintained for interpretability. Each image is first processed with a Gaussian blur to reduce noise and enhance structural consistency, then paired with its corresponding encoded label, enabling supervised multi-class classification. This structured encoding ensures consistent label assignment across the dataset and supports efficient integration within the multi-task learning framework.

### Multi-task architecture

**Fig. 6:**
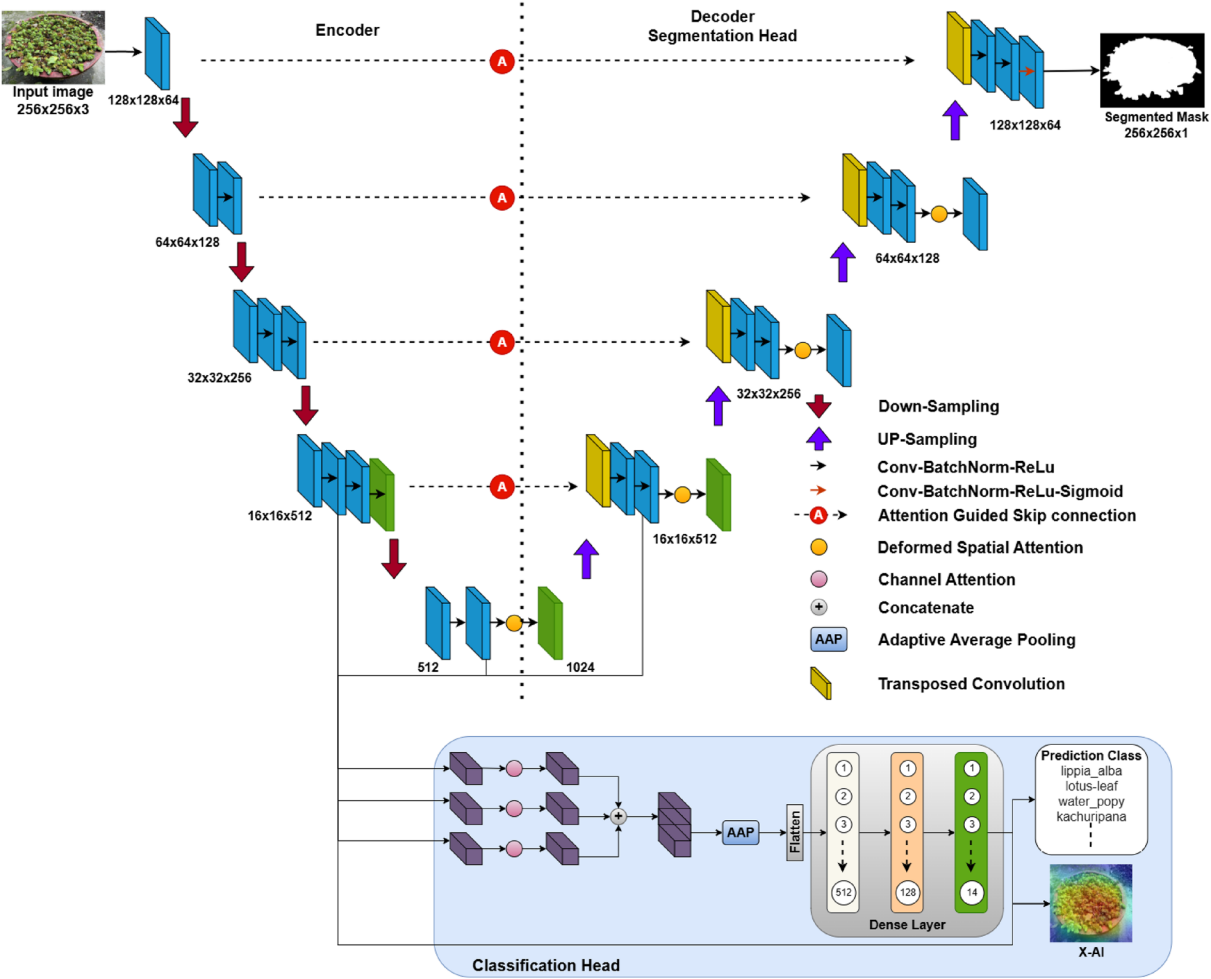
Overview of the proposed attention-enhanced multi-task U-Net. A shared encoder extracts hierarchical feature maps, which are passed through attention-gated skip connections into the segmentation decoder, while a bottleneck-based head performs 14-class species classification from globally pooled features.

The proposed network consists of a shared encoder and task-specific decoders for segmentation and classification. The encoder is a four-stage convolutional backbone that progressively downsamples the input and stores intermediate feature maps for skip connections, while the decoder reconstruct dense masks and predict image-level class labels from the shared representation. Fig. 6 provides an overview of the attention-enhanced multi-task U-Net, illustrating the common encoder, attention-gated skip connections, and the separation between the segmentation decoder and the classification head. The joint design encourages the encoder to capture features that are simultaneously useful for delineating plant regions and discriminating among visually similar aquatic species.

#### Encoder

At each stage $l$, a DoubleConv block applies two consecutive $3 \times 3$ convolutions with batch normalization and ReLU activation:

2 $$\begin{aligned} \textbf{y}^{(l)} = \phi \big (\textrm{BN}(\textrm{Conv}_{3 \times 3}(\phi (\textrm{BN}(\textrm{Conv}_{3 \times 3}(\textbf{x}^{(l)})))))\big ), \end{aligned}$$

where $\phi (\cdot )$ is ReLU. Max pooling with kernel 2 and stride 2 produces the next-scale feature map $\textbf{x}^{(l+1)}$. The encoder outputs a bottleneck feature tensor $\textbf{B} \in \mathbb {R}^{512 \times 16 \times 16}$ and a list of residual feature maps $\{\textbf{R}_l\}$ for skip connections.

#### Attention-guided skip connections

Each skip connection is modulated by an AttentionBlock that gates the encoder feature $\textbf{R}_l$ using the corresponding decoder gate feature $\textbf{G}_l$. The block first projects both features into a low-dimensional space:

3 $$\begin{aligned} \textbf{g}_l = W_g * \textbf{G}_l,\quad \textbf{x}_l = W_x * \textbf{R}_l, \end{aligned}$$

where $W_g$ and $W_x$ are $1 \times 1$ convolutions. The attention coefficients are computed as

4 $$\begin{aligned} \psi _l = \sigma \big (W_\psi * \phi (\textbf{g}_l + \textbf{x}_l)\big ), \end{aligned}$$

with $W_\psi$ a $1 \times 1$ convolution, $\phi$ ReLU, and $\sigma$ the sigmoid function. The attended skip feature is

5 $$\begin{aligned} \tilde{\textbf{R}}_l = \psi _l \odot \textbf{R}_l, \end{aligned}$$

where $\odot$ denotes element-wise multiplication.

#### Segmentation decoder

The segmentation decoder applies an initial DoubleConv that expands the bottleneck from 512 to 1024 channels to increase capacity for spatial reconstruction. It then performs four upsampling stages, where at each stage a transposed convolution is used to double the spatial resolution of the feature map, the corresponding encoder feature map is modulated through an attention gating mechanism, and the upsampled feature is concatenated with the attended skip feature along the channel dimension before being refined by a DoubleConv block.

Formally, at stage $l$:

6 $$\begin{aligned} \begin{aligned} \textbf{U}_l&= \textrm{Up}(\textbf{F}_{l+1}), \quad \tilde{\textbf{R}}_l = \textrm{Attn}(\textbf{U}_l, \textbf{R}_l), \\ \textbf{F}_l&= \textrm{DoubleConv}([\textbf{U}_l, \tilde{\textbf{R}}_l]). \end{aligned} \end{aligned}$$

where $[\cdot ,\cdot ]$ denotes channel-wise concatenation. A final $1 \times 1$ convolution produces a single-channel logit map $\hat{\textbf{M}} \in \mathbb {R}^{1 \times H \times W}$.

#### Classification decoder

For the *ClassificationUNet*, a symmetric attention-based decoder is used to exploit spatial structure before global pooling. The bottleneck is first expanded to 1024 channels via DoubleConv, followed by four upsampling and attention-fusion stages analogous to the segmentation decoder. The final high-resolution feature map $\textbf{F}^{\text {cls}} \in \mathbb {R}^{64 \times H \times W}$ is aggregated with global average pooling and a two-layer MLP:

7 $$\begin{aligned} \textbf{z} = \frac{1}{HW} \sum _{i,j} \textbf{F}^{\text {cls}}_{:,i,j},\quad \hat{\textbf{y}} = W_2 \phi (W_1 \textbf{z} + \textbf{b}_1) + \textbf{b}_2, \end{aligned}$$

where $\textbf{F}^{\text {cls}}$ denotes the final decoder feature map with 64 channels and spatial dimensions $H \times W$; $\textbf{F}^{\text {cls}}_{:,i,j}$ represents the feature vector at spatial location (*i*, *j*); $\textbf{z} \in \mathbb {R}^{64}$ is the global feature vector obtained via global average pooling; $W_1 \in \mathbb {R}^{d \times 64}$ and $W_2 \in \mathbb {R}^{14 \times d}$ are learnable weight matrices; $\textbf{b}_1 \in \mathbb {R}^{d}$ and $\textbf{b}_2 \in \mathbb {R}^{14}$ are bias vectors; $\phi (\cdot )$ denotes a nonlinear activation function (e.g., ReLU); and $\hat{\textbf{y}} \in \mathbb {R}^{14}$ represents the predicted class logits for the 14 aquatic plant species.

For an attention-enhanced multi-task learning model, the shared encoder and segmentation decoder are retained, while classification is performed directly on the bottleneck feature $\textbf{B}$ via adaptive global average pooling and an MLP with 128 hidden units and dropout 0.5. This design encourages the encoder to learn representations that are simultaneously useful for segmentation and classification while keeping the classification head lightweight.

### Loss functions and training

#### Segmentation loss

For binary segmentation, the model outputs logits $\hat{\textbf{M}}$; probabilities are obtained via sigmoid $\textbf{P} = \sigma (\hat{\textbf{M}})$. The Dice loss is used to directly optimize overlap between prediction and ground truth mask $\textbf{M}$:

8 $$\begin{aligned} \mathscr {L}_{\text {Dice}} = 1 - \frac{2 \sum _{i} P_i M_i + \varepsilon }{\sum _{i} P_i + \sum _{i} M_i + \varepsilon }, \end{aligned}$$

where $P_i$ and $M_i$ are flattened pixel values and $\varepsilon$ is a smoothing constant.

#### Classification loss

For 14-class species classification, cross-entropy loss is applied to the logits $\hat{\textbf{y}}$ and ground truth label $y \in \{1,\dots ,14\}$:

9 $$\begin{aligned} \mathscr {L}_{\text {CE}} = - \log \frac{\exp (\hat{y}_y)}{\sum _{k=1}^{14} \exp (\hat{y}_k)}. \end{aligned}$$

where $\hat{\textbf{y}} = [\hat{y}_1, \hat{y}_2, \dots , \hat{y}_{14}] \in \mathbb {R}^{14}$ denotes the predicted logit vector for all classes; $\hat{y}_y$ represents the logit corresponding to the ground truth class index *y*; $\exp (\cdot )$ is the exponential function; the denominator $\sum _{k=1}^{14} \exp (\hat{y}_k)$ computes the normalization term over all class logits; and $\mathscr {L}_{\text {CE}}$ is the categorical cross-entropy loss measuring the discrepancy between predicted logits and the true class label.

#### Multi-task objective

In the joint setting, segmentation and classification losses are combined as

10 $$\begin{aligned} \mathscr {L}_{\text {MTL}} = \lambda _{\text {seg}} \mathscr {L}_{\text {Dice}} + \lambda _{\text {cls}} \mathscr {L}_{\text {CE}}. \end{aligned}$$

In our experiments, equal weighting $\lambda _{\text {seg}} = \lambda _{\text {cls}} = 1$ is used.

#### Optimization

All models are trained using the Adam optimizer with a learning rate of 0.0001 and default parameters $\beta _1 = 0.9$ and $\beta _2 = 0.999$, with a batch size of 32 for both segmentation and classification branches and a maximum of 50 epochs. The search space and final choices for the main optimization-related hyperparameters are summarized in Table 3, which includes optimizer type, learning rate range, candidate batch sizes and epoch budgets, loss weights $\lambda _{\text {seg}}$ and $\lambda _{\text {cls}}$, dropout rate in the classifier MLP, and the hardware configuration (2$\times$T4 GPUs on Kaggle).

During joint training, a unified loop alternates mini-batches from the segmentation and classification dataloaders, computes the corresponding task-specific loss for each batch, and updates the shared encoder and task-specific heads accordingly, while saving checkpoints and best-performing weights for the segmentation-only, classification-only, and multi-task models based on their respective validation metrics. These metrics include Dice coefficient, mean Intersection over Union (mIoU), and pixel accuracy for segmentation, and overall accuracy, macro-averaged precision, recall, F1-score, and confusion matrix for classification.

**Table 3:** Optimization-related hyperparameters.

Hyperparameter name	Space	Selected value
Optimizer	[SGD, Adam, AdamW]	Adam
Learning rate	[0.001, 0.0001, 0.0003, 0.0005]	0.0001
Batch size	[16, 32, 64]	32
Max epochs	[30, 50, 100]	50
Loss weights $\lambda _{\text {seg}}, \lambda _{\text {cls}}$	$[0.5, 1.0, 2.0]$	$1.0, 1.0$
Dropout	$[0.3,0.5, 0.7]$	0.5 (classifier MLP)

### eXplainable Artificial Intelligence (X-AI) Implementation

To improve the interpretability of the proposed multi-task framework, gradient-based class activation mapping is employed to visualize discriminative regions responsible for species classification. Grad-CAM and Grad-CAM++ are applied to the final convolutional layer of the shared encoder, i.e., the bottleneck feature tensor $\textbf{B} \in \mathbb {R}^{C \times H \times W}$ in the attention-enhanced multi-task learning model.

Let $y^c$ denote the logit corresponding to class *c*. For Grad-CAM, the importance weight of the channel *k* in the feature map $\textbf{A}^k$ is computed using global average pooling of gradients:

11 $$\begin{aligned} \alpha _k^c = \frac{1}{Z} \sum _i \sum _j \frac{\partial y^c}{\partial A_{ij}^k}, \end{aligned}$$

where $Z = H \times W$. The class-discriminative localization map is then computed as:

12 $$\begin{aligned} L_{\text {Grad-CAM}}^c = \textrm{ReLU}\left( \sum _k \alpha _k^c \textbf{A}^k \right) . \end{aligned}$$

Grad-CAM++ improves localization by incorporating higher-order gradient information. The channel-wise weights are computed as:

13 $$\begin{aligned} \alpha _k^c = \sum _i \sum _j \frac{ \frac{\partial ^2 y^c}{\partial (A_{ij}^k)^2} }{ 2 \frac{\partial ^2 y^c}{\partial (A_{ij}^k)^2} + \sum _{a,b} A_{ab}^k \frac{\partial ^3 y^c}{\partial (A_{ab}^k)^3} }, \end{aligned}$$

and the corresponding activation map is:

14 $$\begin{aligned} L_{\text {Grad-CAM++}}^c = \textrm{ReLU}\left( \sum _k \alpha _k^c \textbf{A}^k \right) . \end{aligned}$$

Gradients are backpropagated from the classification head to the selected convolutional layer. The resulting heatmaps are upsampled to the input resolution and overlaid on the UAV imagery. In our architecture, attention-gated skip connections enhance the quality of these visualizations by suppressing irrelevant background responses and emphasizing vegetation structures. Grad-CAM provides coarse localization over dominant plant regions, whereas Grad-CAM++ yields sharper and more spatially precise activations aligned with fine morphological details such as leaf clusters and stems. These visual explanations confirm that the proposed multi-task model focuses on ecologically meaningful plant structures, improving transparency and reliability for practical wetland monitoring applications.

The complete training and inference procedure of the proposed architecture, including preprocessing, encoder–decoder operations, attention refinement, loss computation, optimization, and post-hoc interpretability via Grad-CAM and Grad-CAM++, is formally summarized in Algorithm 1.

**Algorithm 1:**
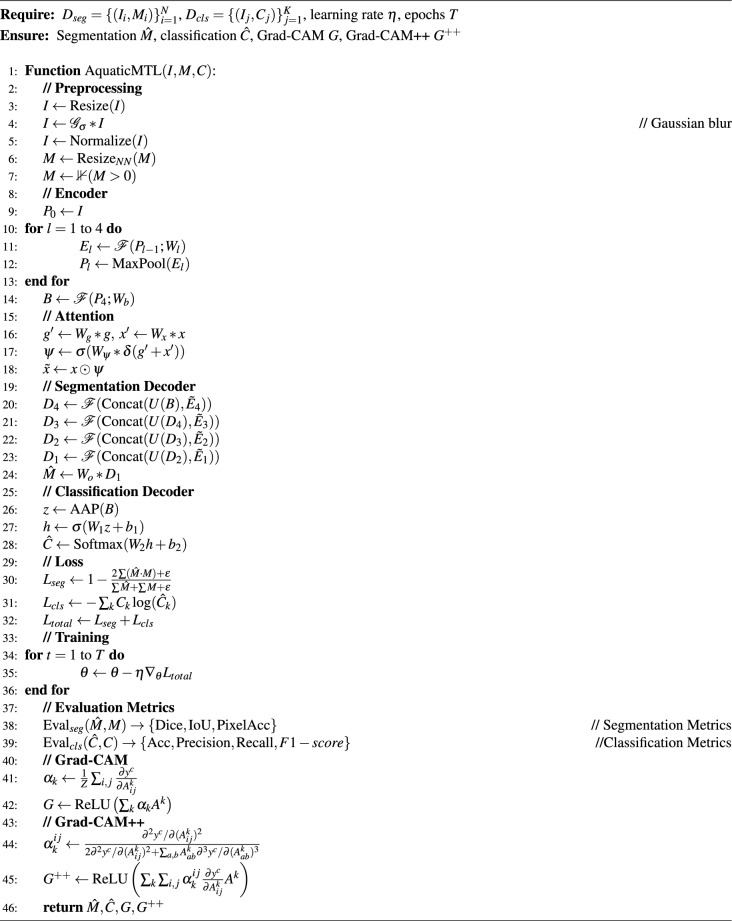
Multi-Task U-Net Framework for Aquatic Plant Segmentation and Classification

## Results analysis

This section presents quantitative results for the proposed models, including single-task and attention-enhanced multi-task learning model performance, followed by an extended ablation study, an explanation of the X-AI results, a comparison with existing work, and a detailed computational complexity analysis.

### Overall single-task and joint performance

Table 4 summarizes the performance of the segmentation-only model, the classification-only model, and the proposed attention-enhanced multi-task model on the aquatic UAV dataset with 14 species classes. The segmentation-only model achieves a Dice coefficient of 0.8591, a mean IoU of 0.8008, and a pixel accuracy of 0.9303, indicating strong capability in capturing plant regions and fine spatial boundaries. This performance is primarily attributed to the model being optimized for a single objective, allowing its full representational capacity to focus on pixel-level discrimination without interference from competing task gradients. However, despite these high quantitative scores, the model lacks semantic awareness of species-level differences, which may limit its effectiveness for comprehensive ecological analysis.

The classification-only model attains 0.7783 accuracy, 0.8154 precision, 0.7721 recall, and 0.7578 F1-score, indicating moderate performance for 14-class species recognition. This result suggests that pure classification benefits from global feature learning; however, the lack of explicit spatial guidance can make the model more susceptible to background variations, water reflections, and inter-class similarity among morphologically related species. In contrast, the proposed attention-enhanced multi-task model achieves 0.7344 Dice, 0.6904 mIoU, and 0.8757 pixel accuracy for segmentation, alongside 0.9877 classification accuracy and 0.9874 F1-score. While segmentation performance decreases compared to the segmentation-only baseline ($-0.12$ Dice), classification performance improves substantially ($+0.21$ accuracy and $+0.23$ F1-score) compared to the classification-only model.

**Table 4:** Overall single-task and joint performance on the aquatic UAV dataset.

Model	Task(s)	Dice	mIoU	Pixel accuracy	Accuracy	Precision	Recall	F1-score
Segmentation-only	Binary seg.	0.8591	0.8008	0.9303	–	–	–	–
Classification-only	14-class cls.	–	–	–	0.7783	0.8154	0.7721	0.7578
Joint attention MTL	Seg. + cls.	0.7344	0.6904	0.8757	0.9877	0.9872	0.9883	0.9874

This trade-off highlights a key characteristic of multi-task learning: shared representations prioritize globally discriminative features that benefit classification, sometimes at the cost of fine-grained boundary precision required for segmentation. The reduction in segmentation accuracy can be attributed to feature competition within the shared encoder, where high-level semantic abstraction for classification reduces sensitivity to small object boundaries and thin plant structures. At the same time, the substantial gain in classification performance is driven by the integration of pixel-level structural cues from the segmentation branch, which helps the model focus on plant regions and suppress irrelevant background features. Attention-gated skip connections further enhance this effect by filtering noise and reinforcing biologically meaningful regions, leading to near-perfect class separability.

**Fig. 7:**
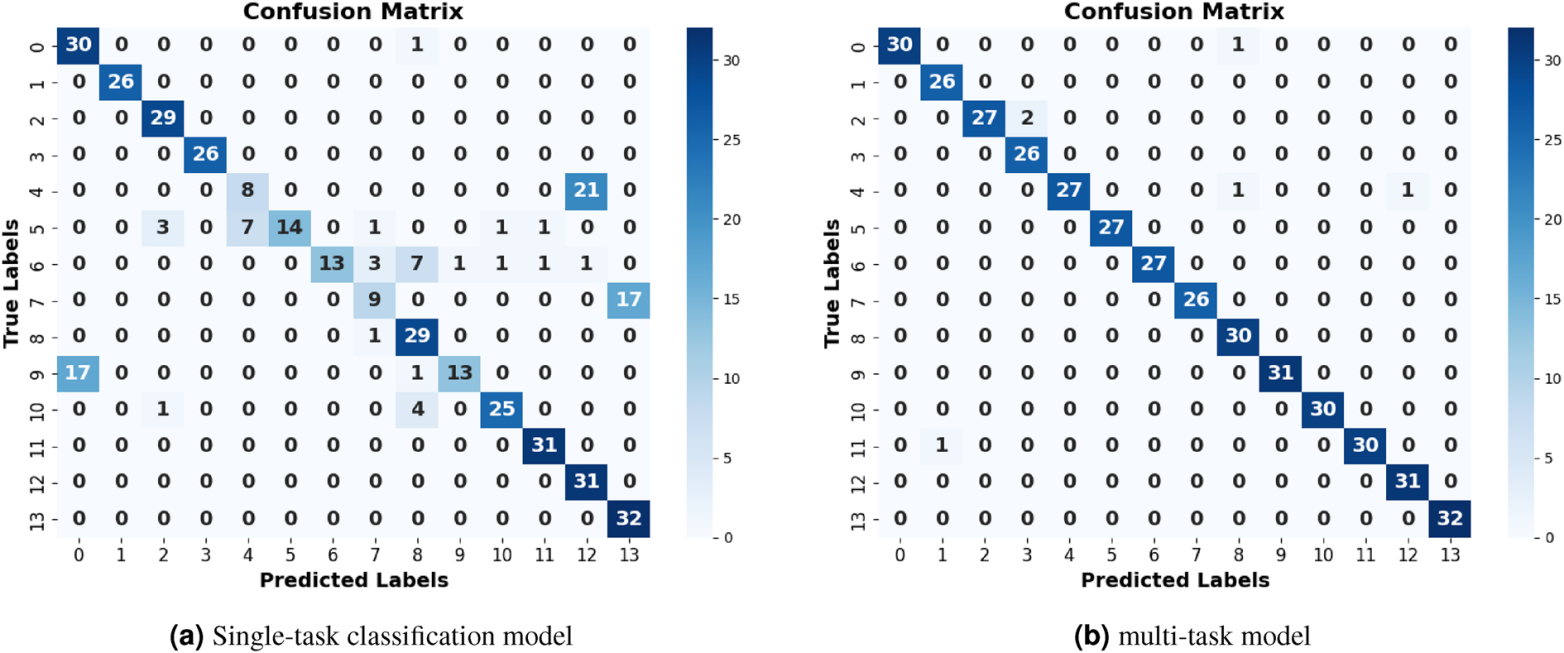
Confusion matrices for 14-class aquatic plant classification: (a) single-task classification model and (b) proposed attention-enhanced multi-task model. The MTL model shows stronger diagonal dominance and reduced inter-class confusion compared to the single-task baseline.

Fig. 7 provides a detailed comparison of class-wise prediction behavior. The single-task classification model (Fig. 7a) exhibits noticeable inter-class confusion, particularly among visually similar aquatic species. Significant off-diagonal values indicate misclassification between classes such as Chinese Water Spinach (Class 6), Water Plantain (Class 7), and other floating vegetation types, reflecting the limitation of relying solely on global image features. Additionally, background interference and water reflections contribute to inconsistent predictions across several classes.

In contrast, the proposed attention-enhanced multi-task model (Fig. 7b) demonstrates strong diagonal dominance with near-perfect classification for most classes, including Water Iris (Class 0), Pistia Stratiotes (Class 1), Mexican Sword Lily (Class 8), Marsilea Minuta (Class 10), Duck Weeds (Class 11), and Topapana (Class 12). The drastic reduction in off-diagonal entries confirms that segmentation-guided feature learning significantly improves class discrimination. Only minor confusion persists in a few categories, such as Water Poppy (Class 4), where visual similarity and overlapping plant structures still pose challenges. The improvement in classification accuracy is therefore not incidental but directly linked to the multi-task design, where segmentation acts as an implicit attention mechanism that localizes relevant regions and enhances feature separability. Conversely, the slight degradation in segmentation reflects the balancing act between spatial precision and semantic abstraction inherent in shared encoder architectures.

**Fig. 8:**
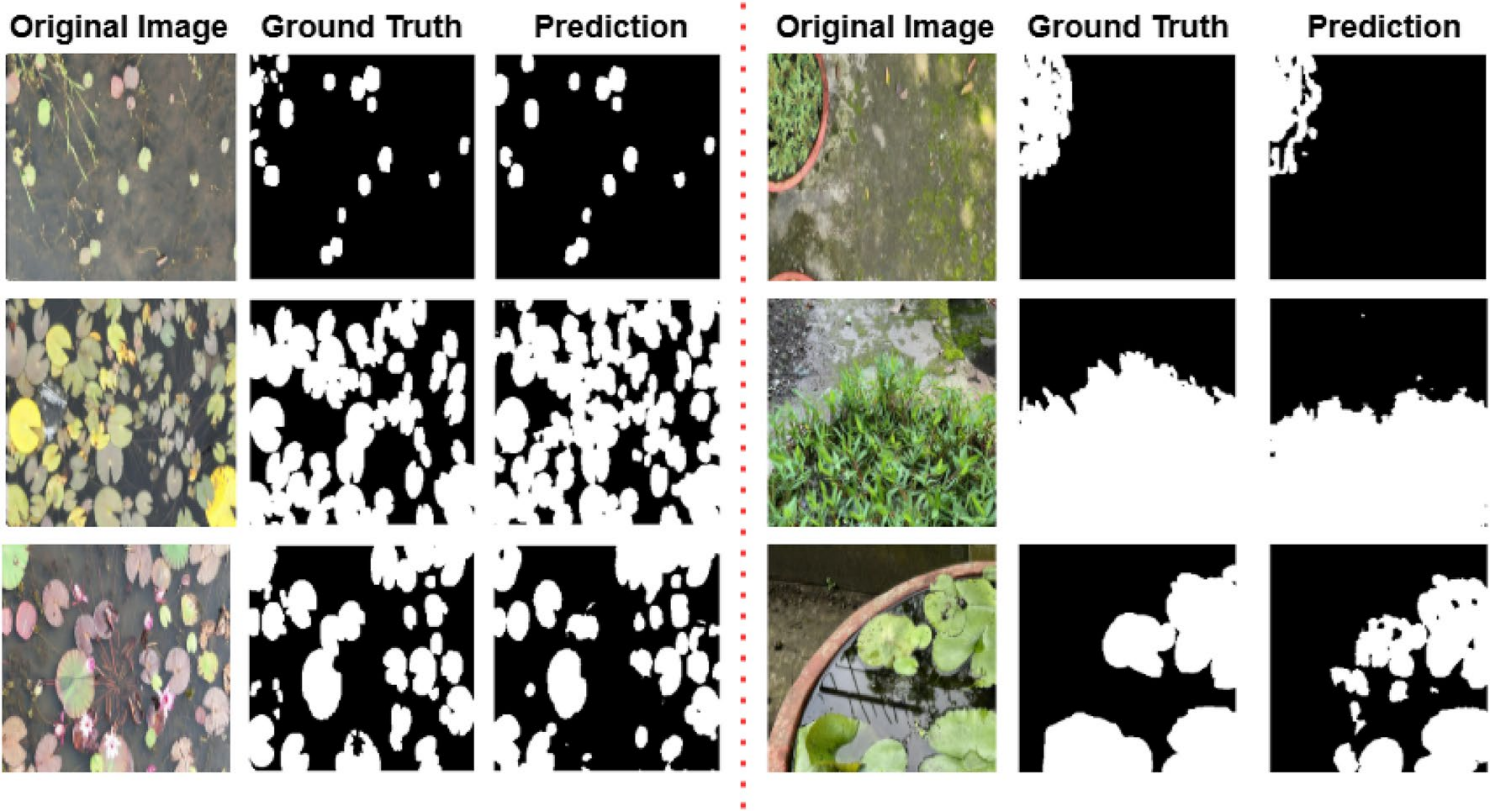
Visual comparison of binary segmentation results, illustrating original UAV images, corresponding ground-truth masks, and predicted masks generated by the multi-task model across varying scene complexities.

Qualitative segmentation results in Fig. 8 demonstrate that the multi-task model effectively handles a wide range of scene complexities, including sparse vegetation, densely clustered floating plants, and heterogeneous environments with mixed object sizes. The model accurately detects small and isolated plant regions while maintaining strong consistency in dense areas, successfully capturing the overall structure of overlapping vegetation. It also performs well in challenging conditions with background noise such as water reflections and soil, although minor false positives and slight boundary smoothing can be observed, particularly in highly crowded or low-contrast regions. Overall, the results indicate that the model achieves a good balance between small-object sensitivity and large-region coherence, producing segmentation outputs that closely align with the ground truth across diverse scenarios.

Overall, the proposed model demonstrates a favorable trade-off: sacrificing a limited amount of segmentation precision to achieve a substantial and practically valuable improvement in fine-grained species classification. This makes the framework highly suitable for real-world ecological monitoring tasks where accurate species identification is often more critical than pixel-perfect boundary delineation.

### Extended ablation study

To comprehensively evaluate the contributions of attention mechanisms, multi-task learning, and input transformations, we conduct an extended ablation study across three dimensions: (i) task configuration (segmentation, classification, and attention-enhanced multi-task model), (ii) image transformation (original RGB vs. Gaussian blur), and (iii) the presence or absence of attention-gated skip connections. The results are summarized in Table 5.

For the segmentation task, models trained on RGB images without attention achieve a Dice score of 0.8626, which improves to 0.8722 when attention is introduced. This gain is consistent with prior findings that attention gates suppress irrelevant background responses and enhance localization of salient regions, particularly in cluttered environments such as water surfaces^25,26^. When Gaussian blur is applied, segmentation performance drops significantly (Dice 0.8116) due to loss of high-frequency spatial details such as edges and fine plant boundaries, which are critical for pixel-level prediction. This behavior aligns with studies showing that edge information is essential for accurate segmentation in natural scenes^27^. However, introducing attention in the blurred setting restores performance to 0.8591 Dice, demonstrating that attention partially compensates for degraded spatial cues by emphasizing structurally consistent regions.

For the classification task, attention provides even more pronounced improvements. On RGB inputs, classification accuracy increases from 0.6207 to 0.8621, and F1-score from 0.5329 to 0.8364 when attention is enabled. This substantial gain occurs because attention mechanisms help the network focus on discriminative plant regions while suppressing background noise, which is especially important in fine-grained classification problems with high inter-class similarity^28,29^. Under Gaussian blur, performance decreases (accuracy 0.6182 to 0.7783 with attention) due to reduced texture and color variation, which are key cues for species-level differentiation. Nevertheless, attention again mitigates this degradation by improving feature aggregation and emphasizing global shape cues over fine textures.

In the attention-enhanced multi-task model setting, a more complex interaction emerges. Without attention, the RGB-based multi-task model achieves relatively low segmentation performance (Dice 0.5947) but surprisingly high classification accuracy (0.9754). This indicates that the shared encoder prioritizes global semantic features beneficial for classification, at the expense of spatial precision required for segmentation, a common trade-off in multi-task learning^30^. Introducing attention significantly improves segmentation (Dice 0.7140), confirming that attention helps recover spatial detail by aligning encoder and decoder features across scales. However, classification accuracy drops slightly (0.9754 to 0.9113) because the model redistributes capacity toward spatial feature refinement rather than purely global discrimination.

Under Gaussian blur, the multi-task model without attention achieves Dice 0.6919 and classification accuracy 0.9852, suggesting that blur acts as an implicit regularizer by suppressing noise and forcing the model to rely on coarse structural patterns rather than fine-grained textures. Similar effects have been observed in robustness studies showing that convolutional neural networks tend to rely heavily on texture, and reducing high-frequency information can improve generalization toward shape-based representations^31^. When attention is added, both segmentation (0.7344 Dice) and classification (0.9877 accuracy) reach their best balanced performance. This demonstrates that attention and multi-task learning are complementary: attention refines spatial localization, while joint supervision improves feature generalization across tasks.

**Table 5:** Detailed ablation study over task type, image transformation, and attention mechanism.

Configuration	Image Transform	Attention	Dice	mIoU	Pixel Accuracy	Accuracy	Precision	Recall	F1-score
Segmentation	RGB	No	0.8626	0.7989	0.9302	-	-	-	-
		Yes	0.8722	0.8083	0.9354	-	-	-	-
	Gaussian Blur	No	0.8116	0.7586	0.9135	-	-	-	-
		Yes	0.8591	0.8008	0.9303	-	-	-	-
Classification	RGB	No	-	-	-	0.6207	0.6309	0.6198	0.5329
		Yes	-	-	-	0.8621	0.8415	0.8567	0.8364
	Gaussian Blur	No	-	-	-	0.6182	0.6572	0.6122	0.5763
		Yes	-	-	-	0.7783	0.8154	0.7721	0.7578
Multi-task Model	RGB	No	0.5947	0.6196	0.8446	0.9754	0.9762	0.9763	0.9745
		Yes	0.7140	0.6923	0.8624	0.9113	0.9421	0.9095	0.9072
	Gaussian Blur	No	0.6919	0.6738	0.8646	0.9852	0.9856	0.9855	0.9851
		Yes	0.7344	0.6904	0.8757	0.9877	0.9872	0.9883	0.9874

Overall, the ablation study reveals three key insights. First, attention consistently improves performance across all tasks and input conditions by enhancing feature selectivity and suppressing background interference. Second, Gaussian blur degrades segmentation due to loss of edge information but can stabilize classification by reducing noise and overfitting to textures. Third, multi-task learning introduces a trade-off between spatial precision and semantic abstraction, which can be effectively balanced through attention mechanisms. These findings confirm that the proposed attention-enhanced multi-task framework leverages complementary strengths of spatial and semantic learning, leading to robust performance in complex aquatic environments.

### X-AI result

**Fig. 9:**
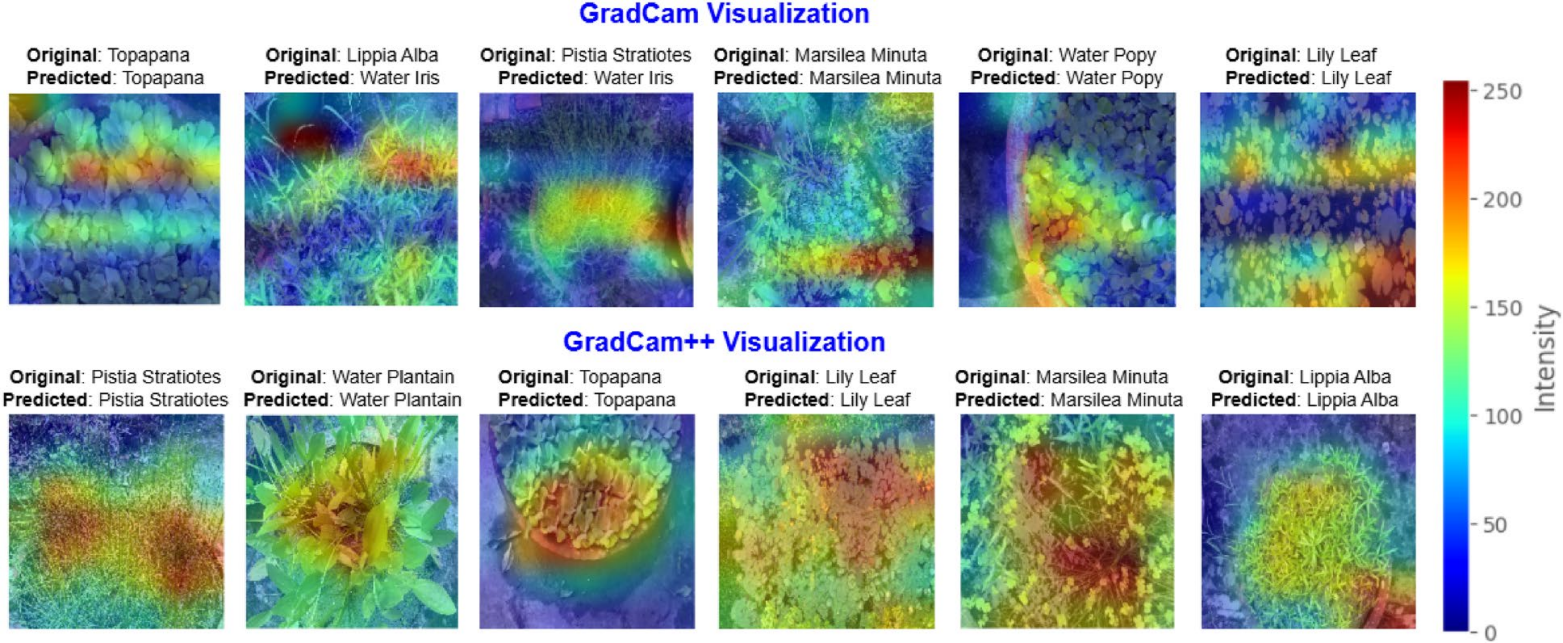
Grad-CAM (top row) and Grad-CAM++ (bottom row) visualizations for representative aquatic plant species. Grad-CAM highlights coarse class-discriminative regions, while Grad-CAM++ produces sharper and more localized activation maps that better align with distinctive morphological structures such as leaf clusters and floral components.

To provide a more rigorous evaluation of model interpretability, Grad-CAM and Grad-CAM++ are employed to analyze class-discriminative regions across both correct and incorrect predictions, as well as multiple plant species. Fig. 9 presents representative examples covering diverse aquatic vegetation types, including floating leaves, grass-like structures, and rosette formations.

From a class-consistency perspective, both Grad-CAM and Grad-CAM++ demonstrate that the model consistently attends to biologically meaningful regions across correctly classified samples. For instance, in species such as Topapana, Marsilea Minuta, and Lily Leaf, the activation maps repeatedly focus on central leaf clusters and characteristic structural arrangements, indicating that the model has learned stable and class-specific visual cues. Grad-CAM++ further refines this behavior by concentrating on finer morphological details such as leaf edges, curvature, and dense cluster cores, suggesting improved localization of discriminative features. A clear distinction emerges when comparing correct versus incorrect predictions. In correctly classified examples (e.g., Water Popy, Lily Leaf, and Marsilea Minuta), both methods highlight coherent plant regions with minimal background interference. In contrast, misclassified cases (e.g., Lippia Alba and Pistia Stratiotes predicted as Water Iris) exhibit more diffuse and fragmented attention maps, where activations partially extend into surrounding vegetation or background textures. This indicates that classification errors are often associated with feature ambiguity and overlapping visual patterns, rather than random model behavior.

Comparing the two methods, Grad-CAM (top row) produces broader and smoother activation regions due to its reliance on global gradient averaging, which can obscure fine-grained structures and occasionally include irrelevant background responses. Grad-CAM++ (bottom row), however, provides more localized and sharper attention, particularly in complex scenes with dense or overlapping vegetation. This improved localization is evident in cases where Grad-CAM++ successfully isolates central plant structures while suppressing noise from water, soil, or container boundaries. Importantly, the visualizations also reveal failure modes of the model. In scenes with high inter-class similarity or cluttered vegetation, the attention maps become less focused, highlighting multiple competing regions. This suggests that errors arise primarily from insufficient discrimination between similar morphological patterns, rather than a lack of attention to relevant regions.

Overall, these results provide stronger evidence that the proposed multi-task model learns semantically meaningful and class-consistent representations, while also exposing its limitations in challenging scenarios. The combination of Grad-CAM and Grad-CAM++ thus offers complementary insights into both the strengths and weaknesses of the model, supporting a more reliable assessment of its interpretability and decision-making behavior.

### Comparison with existing work

The AqUavplant dataset reports U-Net and DeepLabV3 benchmarks achieving up to 85.07% accuracy and 84.11% F1-score-score for binary segmentation under stratified splits, but suffers from severe class imbalance and limited support for joint learning scenarios^32^. Although originally positioned for both segmentation and classification, it primarily provides pixel-level annotations, lacking a standardized image-level classification protocol, which limits its suitability for unified multi-task benchmarking. In contrast, our Aquatic UAV dataset is explicitly designed for dual-task learning, providing balanced annotations for both binary segmentation and 14-class species classification, enabling a fair evaluation of multi-task frameworks. As shown in Table 6, all comparison models are evaluated under a consistent classification protocol derived from segmentation-aware feature extraction, ensuring methodological fairness.

Segmentation-based architectures such as UNet, DeepLabV3, Mask R-CNN, SegFormer, and MSCPUnet are primarily designed for pixel-level prediction and do not directly produce image-level classification outputs. To enable a fair comparison, classification results for these models are generated using a standardized pipeline commonly adopted in remote sensing and medical imaging literature. Following prior approaches^33,34^, global average pooling is applied to deep feature maps extracted from the final encoder or decoder layers, and the resulting representations are passed through a shared lightweight MLP classifier trained under the same supervision as the proposed model. This unified evaluation strategy ensures that classification performance reflects the quality of learned feature representations rather than differences in architectural design. The comparative results in Table 6 show that the proposed model achieves the best overall balance across both tasks, with Dice 0.7344 and mIoU 0.6904, outperforming classical CNN-based segmentation networks such as U-Net^27^ and DeepLabV3^34^. The improvement is attributed to the attention-gated skip connections, which enhance boundary preservation and suppress background noise in highly heterogeneous aquatic scenes. Compared to Mask R-CNN^33^, which introduces instance-level reasoning, our model still achieves superior segmentation consistency due to dense pixel-level supervision without proposal fragmentation, which is particularly beneficial in overlapping vegetation regions.

For classification, the proposed method achieves 0.9877 accuracy and 0.9874 F1-score, significantly outperforming all baseline architectures. This improvement is not due to architectural unfairness but arises from the explicit coupling of segmentation-guided spatial priors with classification features, which helps isolate plant regions before global aggregation. In contrast, transformer-based SegFormer^35^, while strong in classification (0.9631 accuracy), exhibits weaker segmentation performance (Dice 0.6496), highlighting the trade-off between global context modeling and fine spatial precision. The relatively lower classification performance of traditional segmentation networks (e.g., U-Net and DeepLabV3) is mainly due to their reliance on local receptive fields and lack of global semantic pooling mechanisms, making them less effective for fine-grained species discrimination under high inter-class similarity. Meanwhile, Mask R-CNN benefits from region-based learning but suffers from region proposal fragmentation in dense aquatic vegetation, which reduces consistency across overlapping plant structures.

Overall, the comparison in Table 6 is conducted using the same Aquatic UAV dataset for both training and evaluation across all models, ensuring consistency in data distribution and experimental conditions. All models are evaluated under a uniform classification extraction strategy and identical training supervision, ensuring fairness across architectures. The results demonstrate that the proposed attention-enhanced multi-task framework effectively bridges the gap between segmentation-centric and classification-centric learning, achieving strong performance in both tasks through shared representation learning and attention-guided feature refinement.

**Table 6:** Performance comparison of the proposed attention-enhanced multi-task model with existing segmentation and classification methods on the Aquatic UAV dataset.

Model	Segmentation			Classification			
	Dice	mIoU	Pixel Accuracy	Accuracy	Precision	Recall	F1-score
UNet^27^	0.6134	0.6336	0.8394	0.8202	0.8668	0.8158	0.7928
DeepLab V3^34^	0.5546	0.5840	0.8096	0.8818	0.8998	0.8824	0.8767
Mask RCNN^33^	0.7252	0.6848	0.8797	0.9077	0.9001	0.9068	0.9074
SegFormer^35^	0.6496	0.5907	0.7930	0.9631	0.9700	0.9621	0.9641
MSCPUnet^36^	0.6650	0.6557	0.8488	0.8990	0.9274	0.8989	0.8772
**Ours**	0.7344	0.6904	0.8757	0.9877	0.9872	0.9883	0.9874

### Computational complexity analysis

Computational complexity analysis is a critical aspect of evaluating the efficiency of a deep learning model, as it provides insights into the resource requirements during training and inference. Table 7 compares the computational efficiency of the proposed multi-task learning framework with single-task baselines in terms of parameter count, FLOPs, inference time, and GPU memory usage. The results demonstrate that deploying segmentation-only and classification-only models independently leads to a substantial increase in computational cost. When both models are used separately, the total cost reaches 62.09M parameters, 96.31 GFLOPs, 49.38 ms inference time, and 3.83 GB GPU memory usage.

In contrast, the proposed multi-task model significantly improves efficiency by sharing a common encoder while using lightweight task-specific heads, thereby reducing redundancy. The MTL framework requires only 31.10M parameters, achieving approximately a **50%** reduction compared to the combined single-task setup. Similarly, computational complexity is reduced to 54.66 GFLOPs, which is about **1.76**$\times$ lower, while inference latency decreases to 25.38 ms, making it approximately **48.6%** faster. GPU memory usage is also reduced to 2.09 GB, showing a substantial improvement in resource efficiency.

Overall, these results confirm that the proposed architecture effectively balances performance and efficiency by eliminating duplicated feature extraction and leveraging shared representations, making it well-suited for real-time UAV-based applications and deployment in resource-constrained environments.

**Table 7:** complexity comparison between single-task models and the proposed multi-task framework.

Model Configuration	Parameters (M)	FLOPs (GFLOPs)	Inference Time (ms)	GPU Memory (GB)
Segmentation-only	31.04	48.15	24.26	1.86
Classification-only	31.05	48.15	25.12	1.97
**Separate Models (Combined)**	**62.09**	**96.31**	**49.38**	**3.83**
**Proposed MTL (Ours)**	**31.10**	**54.66**	**25.38**	**2.09**

## Discussion

This study introduces an attention-enhanced multi-task learning framework for simultaneous binary segmentation and fine-grained aquatic plant classification from UAV imagery, where pixel-level structural supervision is jointly optimized with image-level semantic understanding through a shared encoder and attention-guided decoding. As shown in Table 4, the proposed attention-enhanced multi-task model reveals an inherent trade-off between the two tasks. Compared to the segmentation-only baseline, segmentation performance decreases (Dice: 0.8591 to 0.7344, mIoU: 0.8008 to 0.6904, Pixel Accuracy: 0.9303 to 0.8757), while classification performance improves substantially (Accuracy: 0.7783 to 0.9877, F1-score: 0.7578 to 0.9874). The slight degradation in segmentation can be attributed to the shared encoder, which must simultaneously learn both fine-grained spatial localization and high-level semantic discrimination, reducing its ability to fully specialize in boundary refinement. In contrast, the dramatic improvement in classification performance stems from the integration of segmentation-aware structural priors, which help the model focus on relevant vegetation regions, suppress background noise, and learn more discriminative features for species-level recognition. This demonstrates that multi-task learning introduces a controlled compromise in dense prediction while significantly enhancing semantic understanding.

The ablation study (Table 5) further confirms the complementary roles of Gaussian blur, attention mechanisms, and joint optimization. Removing attention modules leads to noticeable degradation in both segmentation and classification, indicating that attention improves feature refinement and cross-scale information flow. Similarly, removing the multi-task setting weakens the interaction between tasks, reducing the benefits of shared representation learning. These findings highlight that attention-enhanced feature sharing not only improves spatial-semantic alignment but also strengthens ecologically meaningful representations. Furthermore, comparison with existing methods (Table 6) shows that the proposed framework achieves the most balanced performance across both segmentation and classification, making it more suitable for real-world integrated analysis.

From a computational perspective, the proposed framework remains efficient despite performing two tasks simultaneously. As reported in Table 7, deploying separate segmentation and classification models requires 62.09M parameters, 96.31 GFLOPs, 49.38 ms inference time, and 3.83 GB GPU memory, reflecting significant redundancy due to duplicated encoders. In contrast, the proposed multi-task model reduces the parameter count to 31.10M (approximately 50% reduction), lowers computational cost to 54.66 GFLOPs, and achieves an inference time of 25.38 ms, which is comparable to single-task models (24.26 ms and 25.12 ms) while being nearly twice as fast as the combined setup. GPU memory usage is also reduced to 2.09 GB. Theoretically, this efficiency gain arises because the dominant convolutional operations in the encoder, typically $\mathscr {O}(HWCk^2)$ per layer, are executed only once, while the classification head and attention modules introduce only minimal overhead. This demonstrates that the proposed model effectively eliminates redundant computation while maintaining strong performance, making it well-suited for real-time UAV deployment.

Despite these strengths, several limitations remain. Although the dataset is relatively balanced, certain rare species still have fewer samples, which may limit generalization under extreme class imbalance. Additionally, the current framework performs binary segmentation rather than multi-class segmentation at the pixel level, restricting its ability to directly distinguish species spatially. Moreover, variations in illumination, seasonal conditions, and sensor configurations may introduce domain shifts that are not explicitly addressed.

From an application perspective, the proposed framework has strong potential for large-scale aquatic ecosystem monitoring. It enables automated species-level vegetation mapping, supporting biodiversity assessment, invasive species detection, and habitat health monitoring. UAV-based deployment can significantly reduce reliance on manual field surveys while enabling rapid and scalable analysis. Future work will focus on extending the framework to multi-class semantic and instance segmentation, improving domain generalization through adaptation techniques, and integrating transformer-based or hybrid architectures for better contextual modeling. Additionally, incorporating temporal UAV data could enable dynamic monitoring of vegetation changes, while advancing explainability methods could enhance model transparency for ecological decision-making. Furthermore, future work will also explore the integration of plant height information using depth sensing, stereo imaging, or field-based measurements to enhance structural characterization and improve ecological relevance. Overall, this work demonstrates that attention-enhanced multi-task learning provides an effective, efficient, and scalable solution for integrated aquatic vegetation analysis, bridging the gap between structural mapping and species-level understanding in UAV-based environmental monitoring.

## Conclusion

This work presents an attention-enhanced multi-task U-Net framework that jointly performs binary plant–background segmentation and 14-class aquatic species classification from UAV imagery of Bangladeshi wetlands. By sharing a single encoder and coupling an attention-guided segmentation decoder with a lightweight classification head, the model effectively exploits complementary pixel-level and image-level cues, yielding clear gains over single-task baselines. The multi-task model achieves 0.7344 Dice and 0.6904 mIoU for segmentation, while boosting classification accuracy from 0.7783 to 0.9877 and macro F1-score from 0.7578 to 0.9874, demonstrating the strength of the proposed multi-task design under class imbalance and complex backgrounds. A key contribution is the integration of attention-gated skip connections and multi-task optimization on a curated aquatic UAV dataset, showing that species-level supervision and binary masks are highly synergistic for wetland vegetation analysis. In addition, Grad-CAM and Grad-CAM++ visualizations reveal that the model focuses on ecologically meaningful plant structures rather than background water, improving the interpretability and trustworthiness of predictions. Current limitations include moderate segmentation performance compared to ideal binary benchmarks, sensitivity to rare species imbalance, and evaluation restricted to a single geographic region. Future work will extend the framework to multi-temporal and cross-region settings, richer XAI analyses, multiclass and instance-level segmentation, and tighter integration with ecological decision-support systems.

## Data Availability

The datasets generated and/or analysed during the current study are available in the Mendeley data repository, https://data.mendeley.com/datasets/sf49d949n3/1.
